# Serum cytokine profiling analysis for *zheng* differentiation in chronic hepatitis B

**DOI:** 10.1186/s13020-015-0055-8

**Published:** 2015-08-27

**Authors:** Yi-Yu Lu, Yu Zhao, Ya-Nan Song, Shu Dong, Bin Wei, Qi-Long Chen, Yi-Yang Hu, Shi-Bing Su

**Affiliations:** Research Center for Traditional Chinese Medicine Complexity System, Shanghai University of Traditional Chinese Medicine, 1200 Cailun Road, Pudong, Shanghai, 201203 China; Shuguang Hospital, Shanghai University of Traditional Chinese Medicine, 528 Zhangheng Road, Pudong, Shanghai, 201203 China

## Abstract

**Background:**

*Liver-gallbladder dampness-heat* (LGDH) and *liver kidney yin deficiency* (LKYD) syndromes are Chinese medicine (CM) *zhengs* in chronic hepatitis B (CHB) patients. This study aims to investigate the changes in cytokines and their profiles accompanied by different biological responses in LGDH and LKYD in CHB.

**Methods:**

During 2010–2012, a total of 138 morning fasting venous blood samples were obtained from participants in Shuguang Hospital, Shanghai University of Traditional Chinese Medicine in Shanghai, China. First, serum samples from 20 health controls (HCs) and 40 CHB patients (20 LGDH, 20 LKYD) were collected to detect the profiles of cytokines by multiplex biometric ELISA-based immunoassay. Random forest (RF) with a fivefold cross-validation was used to analyze the significant cytokines. Then the significant cytokines were validated using serum samples from an independent cohort of 60 CHB patients (30 LGDH, 30 LKYD) and 18 HCs.

**Results:**

There were different profiles of cytokines in LGDH and LKYD. Twenty-three significantly differentially expressed cytokines were detected, among which three cytokines, interleukin (IL)-17, macrophage inflammatory protein (MIP)-1α, and MIP-1β, with the largest Gini scores were identified by RF, and further evaluated for their significant changes in serum levels. A receiver-operator characteristic analysis revealed that the logistic regression panel could differentiate LGDH from LKYD (*P* < 0.001; AUC = 0.827). A functional pathway analysis showed that cytokine–cytokine receptor interaction, cytosolic DNA-sensing pathway, and chemokine signaling pathway overlapped between LGDH and LKYD, whereas Toll-like receptor signaling pathway, intestinal immune network for IgA production, NOD-like receptor signaling pathway, and Jak-STAT signaling pathway were only enriched in LGDH.

**Conclusions:**

There were characteristic cytokines profiles in LGDH and LKYD with different inflammatory and immune responses. IL-17, MIP-1α, and MIP-1β might be involved in the differentiation of LGDH and LKYD in CHB.

**Electronic supplementary material:**

The online version of this article (doi:10.1186/s13020-015-0055-8) contains supplementary material, which is available to authorized users.

## Background

Hepatitis B virus (HBV) infection is a potentially adverse sequela of chronic liver failure [1]. Recent epidemiological studies and the World Health Organization estimated that more than 240 million people have chronic (long-term) liver infections globally [2]. More than 780,000 people die every year from the acute or chronic consequences of hepatitis B [3]. Moreover, chronic hepatitis B (CHB) increases the risk for development of cirrhosis and hepatocellular carcinoma [1, 4].

Chinese medicine (CM) has been used for the treatment of liver disease in Asia [5]. *Zheng* (also known as CM syndrome) in CM theory discerns the patterns of imbalances within the body and between the body and the environment through analysis of the symptoms and signs of patients. *Zheng* differentiation by clinical observations and CM practitioners’ experiences, are subjective. Clarifying the biological basis of CM *zheng* differentiation would help establish the objective diagnostic criteria for CM [6].

Clinical pathological changes are referred to CM *zhengs*. A *zheng* is not merely a phenotype based on the profile of symptoms and signs, but reflects a functional dynamic process that can “transform” from one category to another. A *zheng* evolves over time with liver damage in CHB. The *excess* syndrome tends to become a *deficiency* syndrome or intermingled *deficiency* and *excess* syndromes. At the same time, with the development of the disease, cytokines, chemokines, and growth factors secreted by immune system cells and other cell types play important roles in viral clearance, infection control, inflammation, regeneration, and fibrosis in CHB [7]. However, the relationships between typical *zhengs* and cytokines remain unclear.

Reductionist approaches are not suitable for research on the scientific basis of CM [8, 9]. In recent years, there has been great interest in searches for possible biomarkers of *zhengs* by high-throughput omic technologies [10]. High-throughput techniques allow simultaneous examination of dozens or hundreds of proteins and analytical tools facilitate information extraction.

**Fig. 1:**
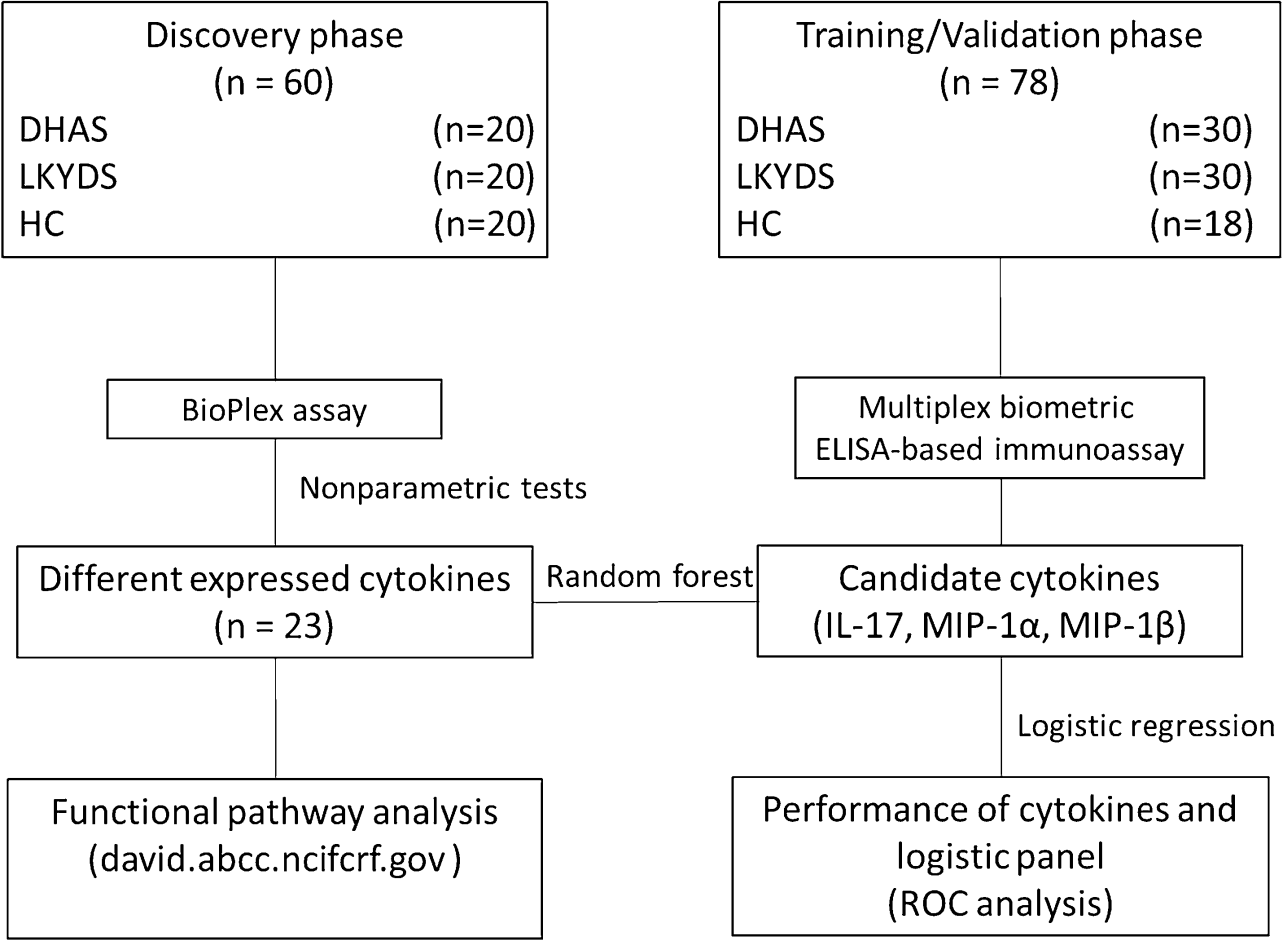
Flow diagram of the study.

Changes in cytokines were reported to be objective indicators of CM *zhengs* [11]. *Liver*-*gallbladder dampness*-*heat* (LGDH) and *liver kidney yin deficiency* (LKYD) syndromes are the two major *zhengs* in CHB [4]. They are typical *zhengs* representing the *excess* syndrome and *deficiency* syndrome, respectively. This study aims to investigate the changes in cytokines and their profiles accompanied by different biological responses in LGDH and LKYD in CHB. We investigated the cytokine profiles in LGDH and LKYD, and the differential expressions of cytokines as potential markers and different biological responses for *zheng* differentiation in CHB (Fig. 1).

## Methods

### Participant recruitment and sample collection

During 2010–2012, a total of 138 morning fasting venous blood samples were obtained from participants in Shuguang Hospital, Shanghai University of Traditional Chinese Medicine in Shanghai, China. Healthy volunteers were recruited in the Physical Examination Center of the Shuguang hospital. First, serum samples from 20 health controls (HCs) and 40 CHB patients (20 LGDH, 20 LKYD) were collected to detect the profiles of cytokines by multiplex biometric ELISA-based immunoassay. Then the significant cytokines were validated using serum samples from an independent cohort of 60 CHB patients (30 LGDH, 30 LKYD) and 18 HCs. The research protocol was approved by the Medical Ethics Committee of Shuguang Hospital (Approval number: 2012-206-22-02; Additional file 1), and informed consent (Additional file 2) was obtained from all study participants. The diagnostic criteria for CHB were based on CHB prevention and treatment guidelines [6]. The *zheng* types were identified according to the guideline for the prevention and treatment of CHB, formulated by the Chinese Society of Hepatology and Chinese Society of Infectious Diseases, Chinese Medical Association (pilot program) [12]. All patients who were diagnosed by attending CM physicians at the first visit and then identified by three chief CM physicians, who generally are the superior of other physicians and usually have over 30 years practice in CM. Those participants who consistently diagnosed as LGDH or LKYD by all three chief CM physicians, were included in the study. Participants with a different diagnosis of any one of the three chief CM physicians were excluded. Patients with other hepatotropic virus hepatitis, chronic severe hepatitis, serious primary disease, or pregnancy were excluded.

### Clinical parameter assessments

Clinical data including age and sex were recorded by a senior physician. Serum levels of total bilirubin (TBIL), direct bilirubin (DBIL), indirect bilirubin (IDBIL), alanine aminotransferase (ALT), aspartate aminotransferase (AST), gamma glutamyl transferase (GGT), alkaline phosphatase (ALP), total protein (TP), albumin (ALB), total bile acid (TBA), Hepatitis B Surface Antigen (HBsAg), Hepatitis B e antigen (HBeAg), and HBV-DNA were measured by an automatic biochemical analyzer (Model LX-20; Beckman, Fullerton, USA). HBsAg, HBeAg, and HBV-DNA were additionally analyzed by an Architect i2000 system (Abbott Laboratories, Abbott Park, IL, USA).

### Multiplex biometric ELISA-based immunoassay using the BioPlex assay and Millipore xMAP assay

Serum samples were collected by centrifugation (Model 3500; KUBOTA, Tokyo, Japan) at 5,700×*g* for 10 min at 4 °C, aliquoted, and stored at −80 °C until analysis. A multiplex biometric ELISA-based immunoassay, containing dyed microspheres conjugated with a monoclonal antibody specific for a target protein, was used according to the manufacturer’s instructions. Soluble molecules were measured using two commercially available kits (BioPlex Assay: M50-0 KCAF0Y, MF0-005KMII; Bio-Plex, Bio-Rad Laboratories Inc., Hercules, CA, USA): (1) 27-Plex panel, including IL-1β, IL-1rα, IL-2, IL-4, IL-5, IL-6, IL-7, IL-8, IL-9, IL-10, IL-12p70, IL-13, IL-15, IL-17, Eotaxin, FGF basic, G-CSF, GM-CSF, IFN-γ, IP-10, MCP-1, MIP-1α, MIP-1β, PDGF-ββ, RANTES, TNF-α, and VEGF; and (2) 21-Plex panel including IL-1α, IL-2Rα, IL-3, IL-12p40, IL-16, IL-18, CTACK, GROα, CXCL9, SDF-1α, HGF, IFNα2, LIF, MCP-3, M-CSF, MIF, β-NGF, SCF, SCGF-β, TNF-β, and TRAIL.

A Millipore xMAP Kit (HCYTOMAG-60K-06; Merck Millipore, Billerica, MA, USA) was applied to detect the serum levels of IL-17, MIP-1α, and MIP-1β in another independent cohort of patients for validation.

Each experiment was performed in duplicate by the same procedure. The serum levels of cytokines were determined by a suspension array (Luminex 200; Luminex, Austin, TX, USA) that quantifies multiplex immunoassays in a 96-well plate with 30-μL aliquots of serum samples. The cytokine concentrations were calculated using a standard curve, with the software provided by the manufacturer.

### Random forest (RF)

RF uses an ensemble of classification trees [13], and returns small sets of independent variables that retain a high predictive accuracy. In this study, we used RF to rank the contribution of each cytokine to discriminate between outcomes of patients with different typical *zhengs*, as a possible index for their biological contributions to the discrimination. The RF method was conducted using the “Random Forest” package [14] with R software (R Foundation for Statistical Computing, version 3.0). A fivefold cross-validation for feature selection of RF was conducted by the “regularized random forest (RRF)” package [15, 16].

Each of the classification trees was built using a bootstrap sample of the data. At each split, the candidate set of variables was a random subset of the total variables. Thus, RF used both bagging (bootstrap aggregation), a successful approach to combining unstable learners, and random variable selection for tree building [17]. Each tree was grown using the classification and regression tree (CART) methodology without pruning. The CART is an iterative classification method for variable selection and prediction of categorical response variables that uses a splitting rule to identify a predictive variable and a cutoff that best breaks the population into homogenous classes. The seed for the random number generator was set to ensure repeatability. In this study, the seed was set at 51. The number of input variables tried for each node was the square root of the number of total variables, and the minimum size of the terminal nodes was set at 2.

### Statistical analysis and functional pathway analysis

All tests for significance were two-sided. Statistical analyses were conducted and false discovery rate-adjusted *P* values were used for multiple comparisons. To compare variables between two groups, the Mann–Whitney U test was applied. To compare variables among multiple groups, the Kruskal–Wallis analysis of variance by ranks test was performed. A stepwise logistic regression model was used to combine diagnostic cytokine markers based on the data obtained in the validation group. The predicted probability of differentiating LGDH from LKYD was used as a surrogate marker to construct a receiver-operating characteristic (ROC) curve. The area under the ROC curve (AUC) was used as an accuracy index for evaluating the diagnostic performance. All tests were two-tailed and *P* values of less than 0.05 were considered statistically significant. A functional pathway analysis was performed using the DAVID online analysis tool (http://www.david.abcc.ncifcrf.gov).

## Results

### Characteristics of the participants

A total of 100 CHB patients (50 LGDH, 50 LKYD) and 38 healthy controls (HCs) were finally enrolled in the study. Forty patients (20 LGDH, 20 LKYD) and 20 HCs were enrolled in the test phase, and 60 patients (30 LGDH, 30 LKYD) and 18 HCs were enrolled in the validation phase (Table 1). The CHB patients were diagnosed as typical LGDH and LKYD and the HC group was derived from healthy volunteers.

**Table 1:** Clinical characteristics of the CHB and HC groups

Parameters	LGDH	LKYD	HC	*P* value (LGDH/LKYD)	*P* value (CHB/HC)
Age (years)	35.3 ± 14.2	36.5 ± 12.2	35.1 ± 18.4	0.654	0.809
Gender (M/F)	18/2	19/1	11/9	0.307	0.432
TBIL (μmol/L)	17.0 ± 7.8	18.1 ± 6.5	15.2 ± 3.6	0.403	0.485
DBIL (μmol/L)	5.3 ± 2.6	4.8 ± 2.0	4.3 ± 0.6	0.650	0.502
IDBIL (μmol/L)	10.9 ± 4.9	13.2 ± 5.3	10.9 ± 3.3	0.131	0.401
ALT (IU/L)	60.9 ± 66.5	66.2 ± 70.8	18.3 ± 6.9	0.721	*0.000*
AST (IU/L)	48.6 ± 32.3	57.4 ± 49.9	18.6 ± 6.0	0.795	*0.000*
GGT (IU/L)	37.8 ± 22.6	33.8 ± 17.4	17.4 ± 6.2	0.506	*0.000*
ALP (IU/L)	95.4 ± 37.7	85.3 ± 25.7	61.2 ± 15.9	0.417	*0.000*
TP (g/L)	75.4 ± 7.1	79.1 ± 5.1	69.5 ± 7.0	0.470	*0.000*
ALB (g/L)	45.0 ± 3.8	46.0 ± 2.9	43.3 ± 3.9	0.548	0.054
TBA (μmol/L)	15.8 ± 24.7	9.4 ± 16.5	8.3 ± 1.9	0.295	*0.006*
HBsAg (IU/mL)	236.9 ± 50.0	236.8 ± 57.4	–	0.780	–
HBeAg (S/CO)	189.7 ± 354.2	422.2 ± 562.3	–	0.380	–
Log HBV-DNA (copies/mL)	6.7 ± 7.3	6.9 ± 7.3	–	0.882	–

Significant *P* values are in italics (*P* < 0.05)

Symbol "–": Health Control group do not have the data of HBsAg, HBeAg and Log HBV-DNA.

The clinical parameters of the patients and HCs enrolled in this study based on the western medical diagnostic approach are shown in Table 1. Compared with the HCs, the levels of ALT, AST, GGT, ALP, TP, and TBA were significantly increased (*P* < 0.01) in the CHB patients. However, the clinical parameters of LGDH and LKYD showed no difference (*P* > 0.05). These parameters could differentiate the CHB patients from the HCs, but were unsuitable for the classification of LGDH and LKYD.

### Comparisons of cytokine levels

The comparisons of the cytokine levels are summarized in Table 2. Among the total of 48 cytokines, the serum concentrations of IL-2, IL-15, Eotaxin, G-CSF, RANTES, GROα, IL-1α, IL-3, IL-12p40, MCP-3, M-CSF, SDF-1α, and TNF-β were beyond the detection range in both groups, and these cytokines were excluded from further analyses. The serum levels of nine cytokines, IL-1β, IL-12, IL-17, FGF basic, IFN-γ, MIP-1α, MIP-1β, TNF-α, and IFN-α2, were significantly differentially expressed between the LGDH group and the LKYD group (*P* < 0.05). Meanwhile, the serum levels of 16 cytokines, IL-1β, IL-8, IL-17, FGF basic, GM-CSF, IFN-γ, IP-10, MIP-1α, PDGF-ββ, VEGF, CTACK, IL-18, LIF, MIG, SCF, and SCGF-β, were significantly differentially expressed for multiple comparisons of the LGDH, LKYD, and HC groups (*P* < 0.05). Two patches of 23 significantly differentially expressed cytokines were merged for subsequent analysis.

**Table 2:** Comparisons of cytokine levels (pg/mL) by the BioPlex assay (mean ± SD)

Cytokines	LGDH (n = 20)	LKYD (n = 20)	HC (n = 20)	*P* value of (LGDH/LKYD)	*P* value of (CHB/HC)
Group I					
IL-1β	2.5 ± 1.5	3.5 ± 1.5	3.4 ± 1.5	*0.011*	*0.016*
IL-1rα	176.9 ± 129.7	246.3 ± 168.1	201.8 ± 159.8	0.065	0.188
IL-2	BDR	BDR	BDR	BDR	–
IL-4	3.1 ± 2.1	3.5 ± 2.0	3.9 ± 2.4	0.101	0.096
IL-5	3.9 ± 1.9	5.0 ± 1.7	4.4 ± 2.1	0.127	0.302
IL-6	9.9 ± 7.6	10.7 ± 8.2	8.2 ± 4.8	0.365	0.578
IL-7	15.6 ± 7.2	15.8 ± 5.9	16.0 ± 7.4	0.089	0.226
IL-8	30.5 ± 18.4	27.1 ± 13.2	48.8 ± 57.4	0.134	*0.042*
IL-9	62.8 ± 103.6	41.1 ± 83.3	8.3 ± 6.8	0.444	0.323
IL-10	10.6 ± 11.7	28.6 ± 92.7	8.8 ± 2.7	0.235	0.340
IL-12 (p70)	21.5 ± 24.8	30.2 ± 50.9	18.6 ± 9.5	*0.035*	0.095
IL-13	12.8 ± 8.1	14.5 ± 7.2	13.1 ± 5.8	0.113	0.208
IL-15	BDR	BDR	BDR	–	–
IL-17	1.6 ± 2.9	4.8 ± 4.5	4.4 ± 3.9	*0.006*	*0.007*
Eotaxin	BDR	BDR	BDR	–	–
FGF basic	17.8 ± 12.8	22.3 ± 12.0	19.7 ± 9.3	*0.019*	*0.040*
G-CSF	BDR	BDR	BDR	–	–
GM-CSF	29.5 ± 32.9	29.0 ± 30.1	71.0 ± 35.2	0.107	*0.003*
IFN-γ	133.4 ± 90.2	150.3 ± 86.8	141.1 ± 108.2	*0.033*	0.086
IP-10	820.7 ± 665.7	1,076.9 ± 733.4	408.9 ± 394.7	0.478	*0.000*
MCP-1 (MCAF)	76.8 ± 46.2	70.3 ± 42.8	67.1 ± 35.4	0.496	0.755
MIP-1α	5.3 ± 2.2	3.6 ± 2.0	5.2 ± 2.4	*0.004*	*0.005*
MIP-1β	154.5 ± 70.4	230.9 ± 101.5	196.3 ± 149.5	*0.026*	0.064
PDGF-ββ	6,536.6 ± 977.7	11,116.8 ± 1,731.4	ADR	0.054	*0.008*
RANTES	ADR	ADR	ADR	–	–
TNF-α	27.7 ± 21.5	26.7 ± 12.9	23.9 ± 12.1	*0.026*	0.110
VEGF	115.7 ± 93.6	90.9 ± 53.1	158.6 ± 82.6	0.749	*0.016*
Group II					
CTACK	1063.6 ± 399.7	1059.6 ± 574.4	606.9 ± 269.0	0.728	*0.002*
GROα	BDR	BDR	BDR	–	–
HGF	940.1 ± 369.3	973.0 ± 424.8	776.9 ± 248.1	0.945	0.375
IFN-α2	145.7 ± 55.0	187.1 ± 49.2	181.9 ± 60.0	*0.047*	0.108
IL-1α	BDR	BDR	BDR	–	–
IL-2Rα	411.0 ± 297.1	414.6 ± 451.6	435.1 ± 431.0	0.945	0.565
IL-3	BDR	BDR	BDR	–	–
IL-12p40	BDR	BDR	BDR	–	–
IL-16	933.7 ± 784.2	968.0 ± 794.5	525.8 ± 335.6	0.627	0.146
IL-18	235.3 ± 99.6	309.7 ± 204.9	166.1 ± 90.1	0.513	*0.022*
LIF	13.2 ± 16.2	15.6 ± 13.2	21.8 ± 12.2	0.089	*0.046*
MCP-3	BDR	BDR	BDR	–	–
M-CSF	BDR	BDR	BDR	–	–
MIF	679.2 ± 548.0	578.1 ± 422.1	587.3 ± 370.4	0.901	0.961
MIG	1,255.3 ± 995.3	1,793.4 ± 1,382.2	900.4 ± 837.2	0.101	*0.044*
β-NGF	2.6 ± 2.3	3.1 ± 2.1	4.8 ± 1.9	0.158	0.085
SCF	215.9 ± 83.7	219.2 ± 82.9	143.7 ± 41.5	0.336	*0.008*
SCGF-β	87,492.2 ± 33,886.1	90,081.1 ± 24,518.4	51,470.7 ± 14,275.2	0.065	*0.002*
SDF-1α	BDR	BDR	BDR	–	–
TNF-β	BDR	BDR	BDR	–	–
TRAIL	209.5 ± 183.4	200.3 ± 143.4	213.0 ± 89.4	0.478	0.443

Significant *P* values are in italics (*P* < 0.05)

*BDR* below the detection range and *ADR* above the detection range. Symbol “–”: Serum concentrations of IL-2, IL-15, Eotaxin, G-CSF, RANTES, GROα, IL-1α, IL-3, IL-12p40, MCP-3, M-CSF, SDF-1α, and TNF-β were beyond the detection range in both groups, thus there were no significant test result of these cytokines.

### LGDH and LKYD classification and candidate cytokine discovery

**Fig. 2:**
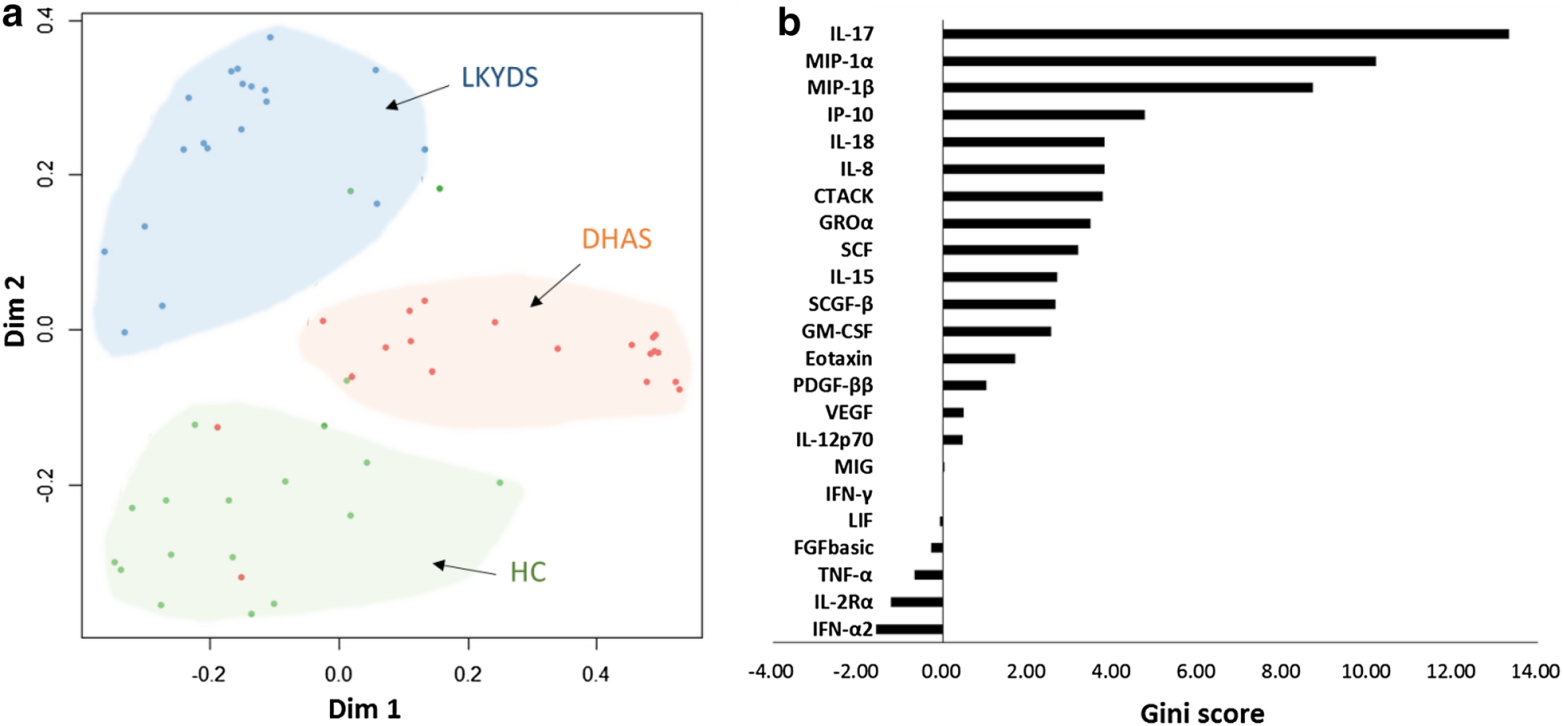
Classification of LGDH, LKYD, and HC groups by cytokines. **a** Plots representing the models discriminating the LGDH, LKYD, and HC groups. The metric multidimensional scaling (MDS) represents the proximity matrices of the RF models, which demonstrate the relationships among the three cohorts. The *two axes* represent the first and second MDS *axes*. *Red dots* LGDH patients; *blue dots* LKYD patients; *green dots* HCs. **b** Relative importance of the cytokines in the overall classification. The *vertical axes* represent the arrangement of individual cytokines according to their importance. The *horizontal axes* represent the average decrease in classification accuracy as the Gini scores. The important cytokines were associated with a greater decrease in classification accuracy.

Twenty-three cytokines were analyzed by RF in the LGDH, LKYD, and HC groups. The results showed a high classification accuracy between the LGDH and LKYD groups (Fig. 2a). The contributions of the individual cytokines for classifying LGDH and LKYD were also calculated. IL-17, MIP-1α, and MIP-1β were found to be required for maximum classification accuracy with first three Gini scores. (Fig. 2b). With 5-fold cross validation (CV) of RF, the model was optimized by selecting the least promising variable with the least CV error. The CV error reached the lowest when the number of variables were two (Additional file 3). Combining both the Gini score and the CV error, we chose three cytokines IL-17, MIP-1α, and MIP-1β for subsequent validation.

### Validation of IL-17, MIP-1α, and MIP-1β selected by RF

**Fig. 3:**
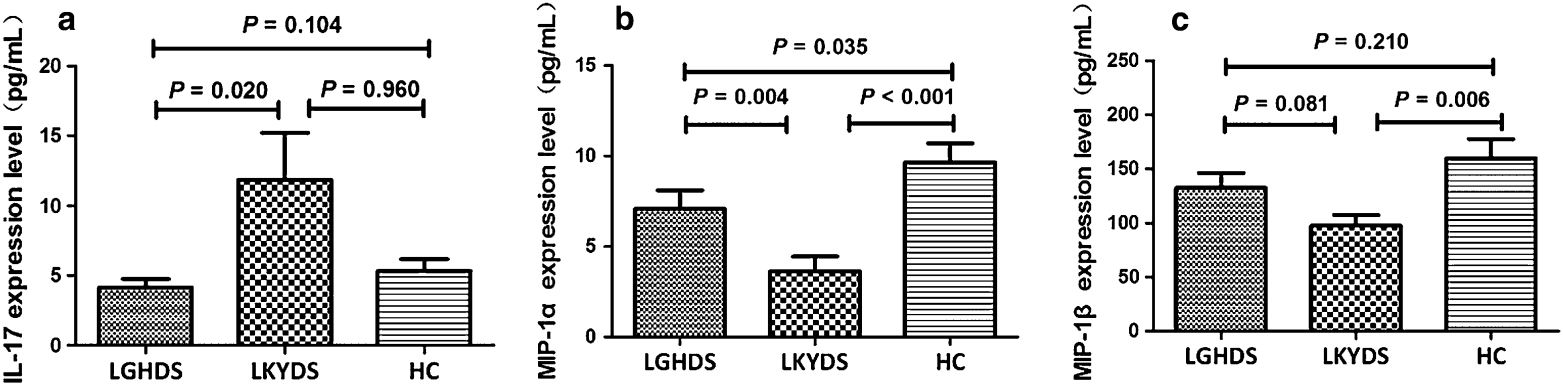
Serum concentrations of candidate cytokines in CHB patients with LGDH or LKYD and in HCs. **a**–**c** Serum levels of IL-17 (**a**), MIP-1α (**b**), and MIP-1β (**c**). LGDH patients, n = 30; LKYD patients, n = 30; HCs, n = 18. Data were analyzed by the Mann–Whitney U test.

Following the computational analyses of RF, an ELISA-based immunoassay was performed on a cohort of independent samples from LGDH patients (n = 30), LKYD patients (n = 30), and HCs (n = 18). The expression results for IL-17, MIP-1α, and MIP-1β were consistent with the results from the multiplex assay. We found statistical significance for these three cytokines (Fig. 3). The IL-17 levels were elevated significantly (*P* = 0.02) in the LKYD group compared with the LGDH group. The MIP-1α levels (*P* = 0.004) were decreased in the LKYD group compared with the LGDH group, while the LGDH and LKYD groups were both remarkably different from the HC group. The MIP-1β (*P* = 0.006) expression levels of LKYDS were significantly different compared with the HC group (Fig. 3). There was no significant difference between the LGDH and LKYD groups (*P* = 0.081).

### Sensitivity and specificity of IL-17, MIP-1α, and MIP-1β for LGDH and LKYD differentiation

**Fig. 4:**
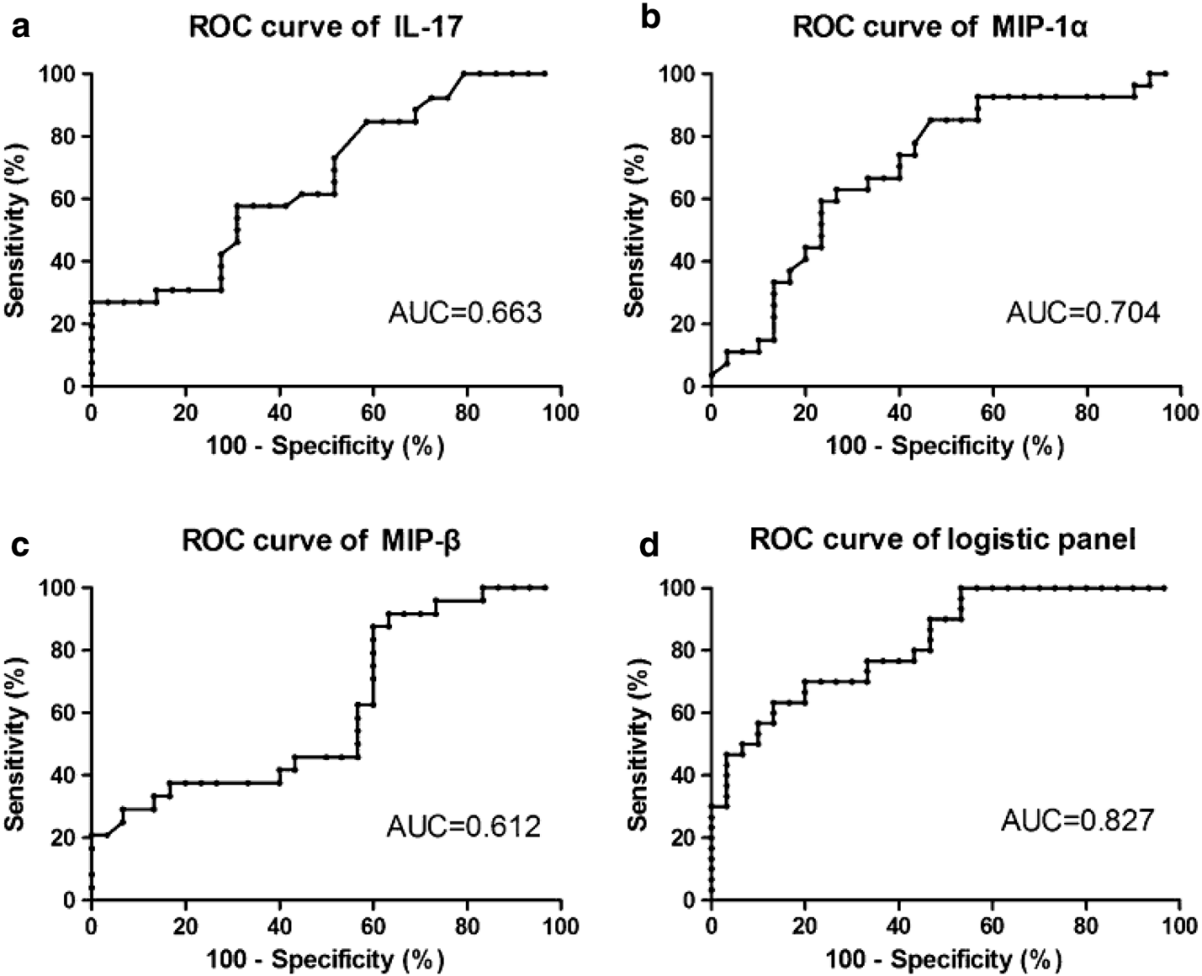
ROC curves for LGDH and LKYD differentiation in CHB. ROC curves were generated by cytokine expression data in CHB patients with LGDH (n = 30) and LKYD (n = 30). **a**–**c** The AUC values for IL-17, MIP-1α, and MIP-1β were 0.663, 0.704, and 0.612, respectively. **d** The AUC value for a logistic panel with the combination of IL-17, MIP-1α, and MIP-1β was 0.827.

The levels of IL-17, MIP-1α, and MIP-1β expressions in LGDH and LKYD were compared. As shown in Fig. 4, the AUC values for IL-17, MIP-1α were 0.663 (*P* = 0.038; 95 % CI 0.5192–0.8058; Fig. 4a), 0.704 (*P* = 0.008; 95 % CI 0.5656–0.8418; Fig. 4b). The AUC for MIP-1β expressions in LGDH and LKYD was 0.612 and showed no significant difference (*P* = 0.16; 95 % CI 0.4574–0.7662; Fig. 4c).

Furthermore, we applied a stepwise logistic regression model to combine the three cytokines for distinguishing between LGDH and LKYD. The logistic model (*P* = LKYD) = 61.50 − 0.402 × IL-17 + 0.336 × MIP-1α + 0.008 × MIP-1β was used to construct the ROC curve. The diagnostic performance for the logistic panel was evaluated by ROC analysis. The AUC for the panel was 0.827 (*P* < 0.001; 95 % CI 0.7256–0.9277; Fig. 4d).

### Functional pathway analysis of significant cytokines in LGDH and LKYD

Cytokine-related signaling pathways were identified by DAVID. Sixteen enriched cytokines differed significantly between LGDH patients and HCs and nine cytokines differed significantly between LKYD patients and HCs. These significant cytokines were then analyzed for further understanding of their biological relevance in LGDH and LKYD. Seven pathways in LGDH were significant at *P* < 0.05 (Table 3). The pathway of cytokine–cytokine receptor interaction showed the most significant *P* values in both the LGDH and LKYD groups, suggesting the strongest association with the input proteins after considering random chance. Toll-like receptor signaling pathway, intestinal immune network for IgA production, NOD-like receptor signaling pathway, and Jak-STAT signaling pathway were only enriched in the LGDH group, while the pathways of cytokine–cytokine receptor interaction, cytosolic DNA-sensing, and chemokine signaling overlapped in the two groups.

**Table 3:** Enriched signaling pathways in LGDH and LKYD

*Zhengs*	Signaling pathways	−log *P* value	Cytokines
LGDH	Cytokine-cytokine receptor interaction	9.41	LIF, IL-4, IL-8, IL-18, IL-1β, IL-15, MIP-1α, CTACK, IP-10
	Cytosolic DNA-sensing pathway	4.02	IL-18, IL-1β, MIP-1α, IP-10
	Toll-like receptor signaling pathway	3.23	IL-8, IL-1β, MIP-1α, IP-10
	Intestinal immune network for IgA production	2.50	IL-4, IL-15, CTACK
	Chemokine signaling pathway	2.46	IL-8, MIP-1α, CTACK, IP-10
	NOD-like receptor signaling pathway	2.30	IL-8, IL-18, IL-1β
	Jak-STAT signaling pathway	1.54	LIF, IL-4, IL-15
LKYD	Cytokine-cytokine receptor interaction	2.58	IL-18, CTACK, IP-10
	Cytosolic DNA-sensing pathway	1.67	IL-18, IP-10
	Chemokine signaling pathway	1.14	CTACK, IP-10

## Discussion

CM *zhengs* such as LGDH (*excess zheng*) and LKYD (*deficiencyzheng*) reflect the two kinds of traditional CM syndromes underlying imbalances in the body. In CM, LGDH is recognized as *dampness*-*heat* accumulation in the *liver* and *gallbladder* resulting in impaired *bile* flow and downward pouring of *dampness*-*heat*, while LKYD is a pathological change in which insufficient *yin fluid* of the *liver* and *kidney* fails to nourish the related body constituents and organs, and gives rise to *deficiency*-*fire* symptoms. The biological validity of these *zhengs* is still unclear, and biological indicators for distinguishing LGDH from LKYD in CHB are also lacking [18]. In chronic viral hepatitis, cytokines modulate a number of critical biological processes, including angiogenesis, neoplastic growth, myofibroblast activation, and responses to viral infections [19, 20], and the cytokine activities lead to the development of fibrosis and cirrhosis [21].

The changes in cytokines in chronic liver disease may be involved in *zheng* differentiation. This study is the first to stratify LGDH and LKYD in CHB using cytokine profiling technology. The results showed that the clinical parameters can easily distinguish CHB patients from HCs, but could not differentiate between the *zhengs* (Table 1). After profiling of cytokines, we found nine cytokines that were differentially expressed between LGDH and LKYD. Sixteen cytokines were differentially expressed for multiple comparisons among the LGDH, LKYD, and HC groups (Table 2).

A multivariate analysis is required to understand the complex relationships between cytokines, and to predict which cytokines can allow discrimination of sample populations. Moreover, we used the RF method to search for potential biomarkers that can distinguish LGDH from LKYD. RF has excellent performance in classification tasks, and has been used in biomarker searches in high-throughput technologies [22]. In this study, there were large Gini scores for IL-17, MIP-1α, and MIP-1β (Fig. 2) for LGDH and LKYD differentiation.

Furthermore, the candidate cytokines of IL-17, MIP-1α, and MIP-1β among the LGDH, LKYD, and HC groups were verified by ELISA-based immunoassay (Fig. 3). In CHB, the differentiation of Th17 cells was promoted by inflammation in liver mesenchymal cells [23]. IL-17 is produced by Th17 cells, and plays a potential role in the amplification of intestinal inflammation stimulating endothelial cells, myofibroblasts, and epithelial cells. Besides, IL-17 was highly expressed in organ fibrosis [24]. In our study, IL-17 was elevated in the LKYD group compared with the LGDH group, which was consistent with the development of chronic liver diseases. MIP-1α and MIP-1β expressions were found in the vascular endothelium of the portal tracts in the normal and HBV-infected liver [25]. MIP-1α and MIP-1β led to increased proliferation and migration of hepatic stellate cells and mediated experimental liver fibrosis [26]. In our study, MIP-1α and MIP-1β were decreased in the LKYD group compared with the LGDH group. The possible reason is that MIP-1α and MIP-1β may have chemoattracted different leukocyte populations toward the inflammatory tissue after continual infection. Therefore, the MIP-1α and MIP-1β levels were decreased in the peripheral blood. LGDH was often observed in the early phase with obvious inflammation in CHB. With the development of CHB, patients show some *deficiency* syndromes such as LKYD or mingled *excess* and *deficiency* syndromes instead of *excess* syndromes. The changes in CM *zhengs* usually follow the development of inflammation in CHB [27].

A ROC curve analysis was conducted to differentiate LGDH and LKYD and observe the sensitivity and specificity of IL-17, MIP-1α, and MIP-1β. AUCs of IL-17 and MIP-1α were significantly different from the null-hypothesis, true area = 0.5 (meaning no discrimination). However, the AUC of MIP-1β was 0.612 and showed weak discrimination ability (*P* = 0.16) (Fig. 4a–c). A larger sample size for validation of MIP-1β was required in the future. The logistic panel with the combination of the three cytokines from the multivariate logistic regression model demonstrated high accuracy in distinguishing between LGDH and LKYD (Fig. 4d). Thus, multiple cytokines should be considered in *zheng* differentiation.

The differentially expressed cytokines were then analyzed for further understanding of their biological significance in LGDH and LKYD. The functional pathway analysis indicated three pathways that overlapped in LGDH and LKYD. All three signaling pathways enriched in LKYD are also enriched in LGDH, which actually contained markedly greater enrichment of Toll-like receptor signaling pathway, intestinal immune network for IgA production, NOD-like receptor signaling pathway, and Jak-STAT signaling pathway. Toll-like receptor signaling pathway and JAK-STAT signaling pathway activations are frequently found in the initial responses to inflammation. They induce the expressions of immune and pro-inflammatory genes [28, 29]. This was consistent with the phenomenon that LGDH is usually observed in the initial stage of CHB and indicates the possibility that LGDH is a *zheng* that develops from LKYD. Toll-like receptors and NOD-like receptors acted as key mediators for chronic liver injury [30]. They were found to be essential for the recognition of invading pathogens and served as important links between innate and adaptive immunity [31, 32]. The activation of JAK/STAT signaling in the liver was associated with increased hepatocyte proliferation in response to stimulation by growth factors or partial hepatectomy [33]. Many tested proteins in these pathways were significantly represented in the setting of LKYD compared with LGDH, suggesting the different pathological states of these *zhengs*. With the activation of these particular enriched pathways, the *zhengs* may evolve from LGDH to LKYD.

All of the above results suggest that LKYD might serve for *zheng*-based treatment according to CM, *i.e.*, “treating *excess* syndrome by purgation and treating *deficiency* syndrome by replenishment”. The successful prediction and selection of biological indicators contribute to the scientific interpretation of CM *zhengs*. In future studies, larger sample sizes and other *zheng* types of CHB should be employed. Because the cytokine regulation networks are complicated and remain unclear in HBV-derived chronic diseases, more cytokines should be tested and the overall mechanism is subject to further evaluation.

## Conclusions

There were characteristic cytokine profiles in LGDH and LKYD with different inflammatory and immune responses. IL-17, MIP-1α, and MIP-1β might be involved in the differentiation of LGDH and LKYD in CHB.

## Additional files

Additional file 1. Approval document of the research protocol by the Medical Ethics Committee of Shuguang Hospital. Additional file 2. Informed consent for the study participants of the research. Additional file 3. The best number of variables with the lowest cross validation (CV) error rate is calculated with 5-fold CV in RF.

## Abbreviations

CM: Chinese medicine
LGDH: *liver*-*gallbladder dampness*-*heat* syndrome
LKYD: *liver kidney yin deficiency* syndrome
CHB: chronic hepatitis B
HCs: healthy controls
RF: random forest
MIP-1: macrophage inflammatory protein-1
HBV: hepatitis B virus
TBIL: total bilirubin
DBIL: direct bilirubin
IDBIL: indirect bilirubin
ALT: alanine aminotransferase
AST: aspartate aminotransferase
GGT: gamma glutamyl transferase
ALP: alkaline phosphatase
TP: total protein
ALB: albumin
TBA: total bile acid
ROC: receiver-operator characteristic
AUC: area under the ROC curve

## References

[1] Lavanchy D (2004). Hepatitis B virus epidemiology, disease burden, treatment, and current and emerging prevention and control measures. J Viral Hepatitis.

[2] Ott J, Stevens G, Groeger J, Wiersma S (2012). Global epidemiology of hepatitis B virus infection: new estimates of age-specific HBsAg seroprevalence and endemicity. Vaccine.

[3] Perz JF, Armstrong GL, Farrington LA, Hutin YJ, Bell BP (2006). The contributions of hepatitis B virus and hepatitis C virus infections to cirrhosis and primary liver cancer worldwide. J Hepatol.

[4] Schütte K, Bornschein J, Malfertheiner P (2009). Hepatocellular carcinoma–epidemiological trends and risk factors. Dig Dis.

[5] Wang X, Sun H, Zhang A, Sun W, Wang P, Wang Z (2011). Potential role of metabolomics apporoaches in the area of traditional Chinese medicine: as pillars of the bridge between Chinese and Western medicine. J Pharm Biomed Anal.

[6] Su S-B, Lu A, Li S, Jia W (2012) Evidence-based ZHENG: a traditional Chinese medicine syndrome. Evid Based Complement Altern Med eCAM 2012:246538 10.1155/2012/246538 PMC3425345 22927876

[7] Meneghin A, Hogaboam CM (2007). Infectious disease, the innate immune response, and fibrosis. J Clin Investig.

[8] Jiang WY (2005). Therapeutic wisdom in traditional Chinese medicine: a perspective from modern science. Trends Pharmacol Sci.

[9] Feng Y, Wu ZH, Zhou XZ, Zhou ZM, Fan WY (2006). Knowledge discovery in traditional Chinese medicine: state of the art and perspectives. Artif Intell Med.

[10] Gertler R, Rosenberg R, Fuehrer K, Dahm M, Nekarda H, Siewert JR (2003) Detection of circulating tumor cells in blood using an optimized density gradient centrifugation. In: Molecular Staging of Cancer. Springer, New York, pp 149–155 10.1007/978-3-642-59349-9_13 12790329

[11] Liang J, Hu L, Zheng X (2012). Study of Th1/Th2 balance in peripheral blood of chronic gastritis patients with Pi-Wei damp-heat syndrome. Chin J Integr Tradit West Med.

[12] Chinese society of Hepatology and Chinese Society of Infectious Diseases CMA (2005). The guideline of prevention and treatment for chronic hepatitis B. Chin J Hepatol.

[13] Breiman L, Friedman J, Stone CJ, Olshen RA (1984). Classification and regression trees.

[14] Liaw A, Wiener M (2002). Classification and Regression by randomForest. R news.

[15] Deng H (2013) Guided random forest in the RRF package. arXiv preprint arXiv:13060237, pp 1–2

[16] Deng H, Runger G (2013). Gene selection with guided regularized random forest. Pattern Recogn.

[17] Hastie T, Tibshirani R, Friedman J, Hastie T, Friedman J, Tibshirani R (2009). The elements of statistical learning.

[18] WHO (2007). WHO international standard terminologies on traditional medicine in the western pacific region.

[19] Germano G, Allavena P, Mantovani A (2008). Cytokines as a key component of cancer-related inflammation. Cytokine.

[20] Racanelli V, Rehermann B (2006). The liver as an immunological organ. Hepatology.

[21] Marra F (2002). Chemokines in liver inflammation and fibrosis. Front Biosci J Virtual Libr.

[22] Saeys Y, Inza I, Larrañaga P (2007). A review of feature selection techniques in bioinformatics. Bioinformatics.

[23] Veldhoen M, Hocking RJ, Atkins CJ, Locksley RM, Stockinger B (2006). TGFβ in the context of an inflammatory cytokine milieu supports de novo differentiation of IL-17-producing T cells. Immunity.

[24] Qian C, Jiang T, Zhang W, Ren C, Wang Q, Qin Q (2013). Increased IL-23 and IL-17 expression by peripheral blood cells of patients with primary biliary cirrhosis. Cytokine.

[25] Shin EC, Choi YH, Kim JS, Kim SJ, Park JH (2002). Expression patterns of cytokines and chemokines genes in human hepatoma cells. Yonsei Med J.

[26] Heinrichs D, Berres M-L, Nellen A, Fischer P, Scholten D, Trautwein C (2013). The chemokine CCL3 promotes experimental liver fibrosis in mice. PLoS One.

[27] Liu Z, Tao X-P, Liu S-N (2010). Study on traditional Chinese medicine syndrome characteristics of chronic hepatitis B. Chin J Basic Med Tradit Chin Med.

[28] Akira S, Takeda K, Kaisho T (2001). Toll-like receptors: critical proteins linking innate and acquired immunity. Nat Immunol.

[29] Kerr IM, Costa-Pereira AP, Lillemeier BF, Strobl B (2003). Of JAKs, STATs, blind watchmakers, jeeps and trains. FEBS Lett.

[30] Fukata M, Vamadevan AS, Abreu MT (2009). Toll-like receptors (TLRs) and Nod-like receptors (NLRs) in inflammatory disorders. Semin Immunol.

[31] Lang T, Lo C, Skinner N, Locarnini S, Visvanathan K, Mansell A (2011). The hepatitis B e antigen (HBeAg) targets and suppresses activation of the toll-like receptor signaling pathway. J Hepatol.

[32] Isogawa M, Robek MD, Furuichi Y, Chisari FV (2005). Toll-like receptor signaling inhibits hepatitis B virus replication in vivo. J Virol.

[33] Cressman DE, Diamond RH, Taub R (1995). Rapid activation of the Stat3 transcription complex in liver regeneration. Hepatology.

