# Supplementary material for: Serum cytokine profiling analysis for zheng differentiation in chronic hepatitis B
Source: Chin Med. 2015 Aug 27;10:24. doi: 10.1186/s13020-015-0055-8 (PMC4550060; doi:10.1186/s13020-015-0055-8)
Supplement: Additional file 1. — Approval document of the research protocol by the Medical Ethics Committee of Shuguang Hospital. [file 13020_2015_55_MOESM1_ESM.pdf]

知情同意书•知情告知页

尊敬的先生/女士：

我们将邀请您参加国家重大科技专项课题（2012ZX10005001-004）“基于转化医学的慢性乙型病毒性肝炎中医证候生物学研究”的研究工作临床试验。

在您决定是否参加这项试验之前，请尽可能仔细阅读以下内容，它可以帮助您了解该项试验以及为何要进行这项试验，试验的程序和期限，参加试验后可能给您带来的益处、风险和不适。如果您愿意，您也可以和您的亲属、朋友一起讨论，或者请您的医生给予解释，帮助您做出决定。

试验介绍

一、试验背景和试验目的

本此项试验方案由曙光医院伦理委员会审议，将遵从赫尔辛基宣言等保护受试者权益的国际原则和中国国家相关法规、规则，符合医疗道德。

本试验的目的是：基于转化医学理念，围绕降低“发病率和病死率”（简称“两率”）的目标，以传染病中医临床治疗课题单位和传染病综合防治示范区为依托，遵循临床流行病学和循证医学原则，引入组学关键技术和方法，深化“十一五”研究成果，明确病毒性肝炎典型证候的生物学标志物（谱），初步阐明传染病典型证候的现代科学内涵，为病毒性肝炎辨证的客观化和疗效评价提供依据，提升中医防治重大传染病的能力和水平。

本试验将在曙光医院、龙华医院、杭州市第六人民医院、绍兴市传染病医院、浙江中医药大学附属新华医院、宁波市第二人民医院等医院进行，预计有1000例受试者自愿参加。

二、哪些人不宜参加试验

如果您：(1)健康人群：年龄不在18-65岁范围；实验室检查结果异常且有临床意义者。（2）慢性乙型肝炎人群：年龄不在18-65岁范围；喜欢吸烟、喝酒；合并其它嗜肝病毒感染的肝炎；慢性重型肝炎和肝硬化；孕妇或哺乳期妇女；患有重大疾病者；不能正确表达自我感受者不宜参加本试验。(3)非酒精性脂肪性肝炎人群：年龄不在18-65岁范围；喜欢吸烟、喝酒；合并嗜肝病毒感染的肝炎；合并糖尿病者；孕妇或哺乳期妇女；患有重大疾病者；不能正确表达自我感受者不宜参加本试验。

三、如果参加试验将需要做什么

您已知道该次临床观察为国家重大项目所需，您志愿参加本次观察，与研究者全面合作。

您将配合医生收集病史，完成四诊信息问卷调查、也将取血液约20ml/次进行实验室检查（肝功能全套、肾功能、血脂、血糖、乙肝病毒标志、HBV-DNA定量、血尿粪便常规、肝纤维化指标、蛋白电泳指标、甲状腺功能等），根据医生要求留取尿液和粪便样本并及时提供给项目组。检查的内容和具体步骤，医生将负责给予说明。

四、参加试验可能的受益

参加此项研究结束时，研究组会在1个月内免费提供临床检查结果报告：包括肝功能全套、肾功能、血脂、血糖、乙肝病毒标志、HBV-DNA定量、血尿粪便常规、肝纤维化指标、蛋白电泳指标、甲状腺功能、肝胆脾肾B超检查等

五、参加试验可能的不良反应、风险和不适、不方便

在签署知情同意的前提下，收集受试者病史，留取血、尿、粪样本，对受试者无直接风

险或危害。

医生和申办者将尽全力预防由于本试验可能带来的伤害。如果在临床试验中出现不良事件，医生将提供有关治疗。

六、有关费用

您将享有一次免费检查的机会，除此之外，本研究不干涉您正常的医疗费用。

如果发生与试验相关的损害，申办者将支付您的医疗费用。如果因严重不良反应住院医疗，申办者还将提供适当的营养费、误工的工资和奖金等补偿费。

七、个人信息是保密的吗？

您的医疗记录（研究病历/CRF、化验单等）将完整地保存在医院。研究者、申办者代表、伦理委员会将被允许查阅您的医疗记录。任何有关本项试验结果的公开报告将不会披露您的个人身份。我们将在法律允许的范围内，尽一切努力保护您个人医疗资料的隐私。

除本试验以外，有可能在今后的其他试验中再次使用您的医疗记录，该医疗纪录将保留除姓名外的其他个人信息。您也可以声明拒绝除本试验外的其他试验使用您的医疗记录和血标本，并将不会因此而受到歧视和报复。

八、怎样获得更多的信息？

您可以在任何时间提出有关本项试验的任何问题。

您的医生将给您留下他/她的电话号码以便能回答您的问题。

如果在试验过程中有任何重要的新信息，可能影响您继续参加试验的意愿时，您的医生将会及时通知您。

九、可以自愿选择参加试验和中途退出试验

是否参加试验完全取决于您的自愿。您可以拒绝参加此项试验，或在试验过程中的任何时间退出本试验。如果您选择退出次研究，您的受益将不会受到影响，也不会因此而受到歧视或报复。

如果您选择参加本项试验，我们希望您能够坚持完成全部试验过程。

十、现在该做什么？

是否参加本项试验由您自己决定。您可以和您的家人或者朋友讨论后再做出决定。

在您做出参加试验的决定前，请尽可能向您的医生询问有关问题，直至您对本项试验完全理解。

感谢您阅读以上材料。如果您决定参加本项试验，请告诉您的医生，他/她会为您安排一切有关试验的事务。

请您保留这份资料。

十一、伦理委员会

如果您有疑问或需要向除研究者、申办者以外的人员询问，请咨询曙光医院伦理委员会

伦理委员会办公室地址： 曙光医院东部行政二楼 伦理委员会办公室

电话：20256070

工作时间：周一～周五　 8：00～17：00

知情同意书•同意签字页

临床试验项目名称：基于转化医学的慢性乙型病毒性肝炎中医证候生物学研究
申办者：中华人民共和国科学技术部
伦理审查委员会：曙光医院伦理委员会

同意声明

我已经阅读了上述有关本试验的介绍，而且有机会就此项试验与医生讨论并提出问题。我提出的所有问题都得到了满意的答复。

我知道参加本试验可能产生的风险和受益。我知晓参加试验是自愿的，我确认已有充足时间进行考虑，而且明白：

- 我可以随时向医生咨询更多的信息。
- 我可以随时退出本试验，而不会受到歧视或报复，医疗待遇与受益不会受到影响。

我同样清楚，如果我中途退出试验，我将病情变化告诉医生，完成相应的体格检查和理化检查，这将对我本人和整个试验十分有利。

我同意伦理委员会或申办者代表查阅我的试验资料。

我将获得一份经过签名并注明日期的知情同意书副本。

最后，我决定同意参加本项试验，并保证尽量遵从医嘱。

我知道除本试验以外的其他试验可能使用我的医疗记录。

同意（签名）________ 不同意（签名）________

受试者签名__________ ________年________月________日

受试者的联系电话：___________________（手机）_____________________（家庭电话）

我确认已向受试者解释了本试验的详细情况，包括其权力以及可能的受益和风险，并给其一份签署过的知情同意书副本。

医生签名：____________ ________年________月________日

医生的工作电话：________________
