# Supplementary figures and images for: Serum cytokine profiling analysis for zheng differentiation in chronic hepatitis B
Source: Chin Med. 2015 Aug 27;10:24. doi: 10.1186/s13020-015-0055-8 (PMC4550060; doi:10.1186/s13020-015-0055-8)

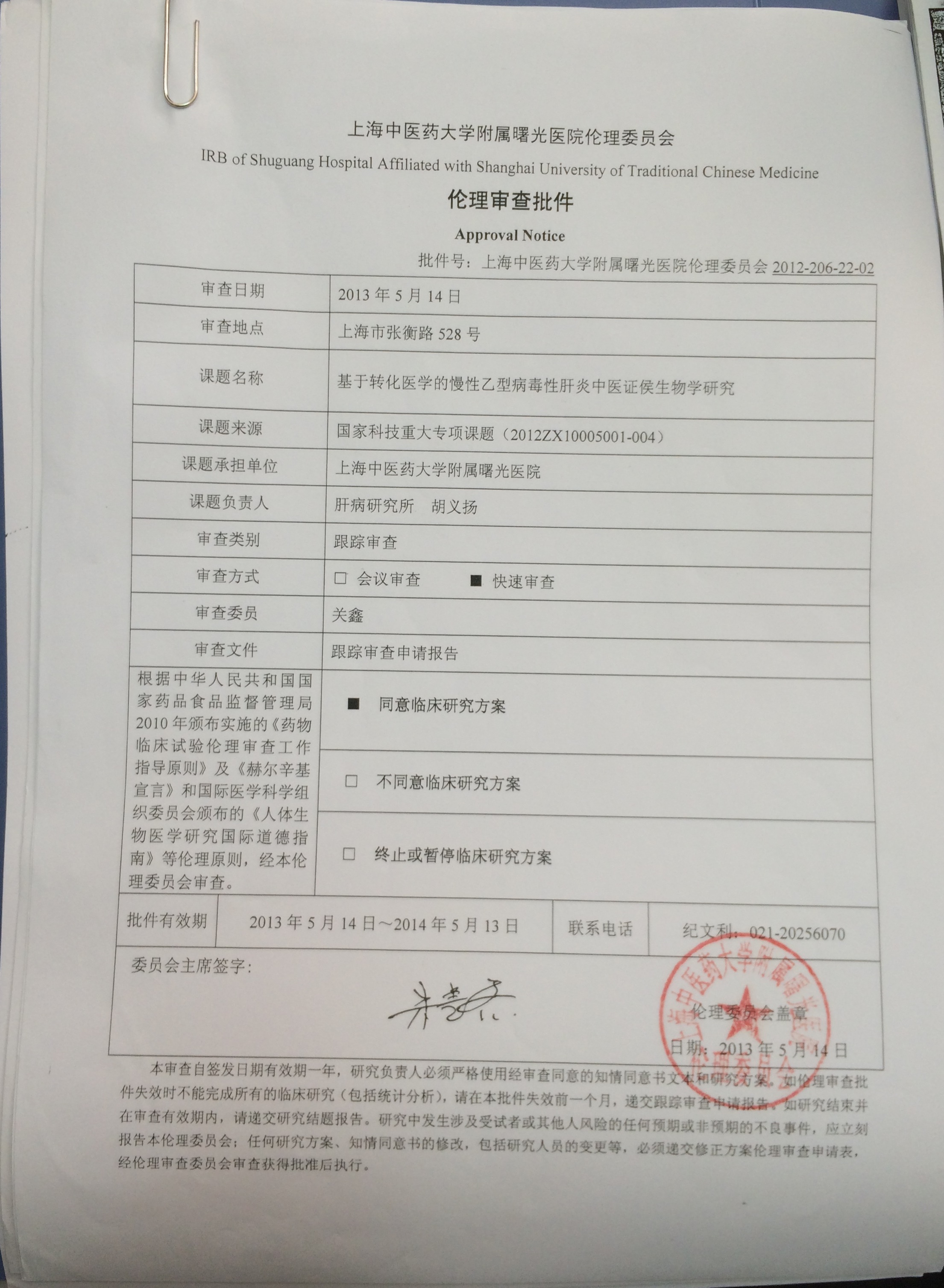

Supplement: Additional file 2. — Informed consent for the study participants of the research. [file 13020_2015_55_MOESM2_ESM.jpeg]
