# Supplementary material for: Serum cytokine profiling analysis for zheng differentiation in chronic hepatitis B
Source: Chin Med. 2015 Aug 27;10:24. doi: 10.1186/s13020-015-0055-8 (PMC4550060; doi:10.1186/s13020-015-0055-8)
Supplement: Additional file 3. — The best number of variables with the lowest cross validation (CV) error rate is calculated with 5-fold CV in RF. [file 13020_2015_55_MOESM3_ESM.pdf]

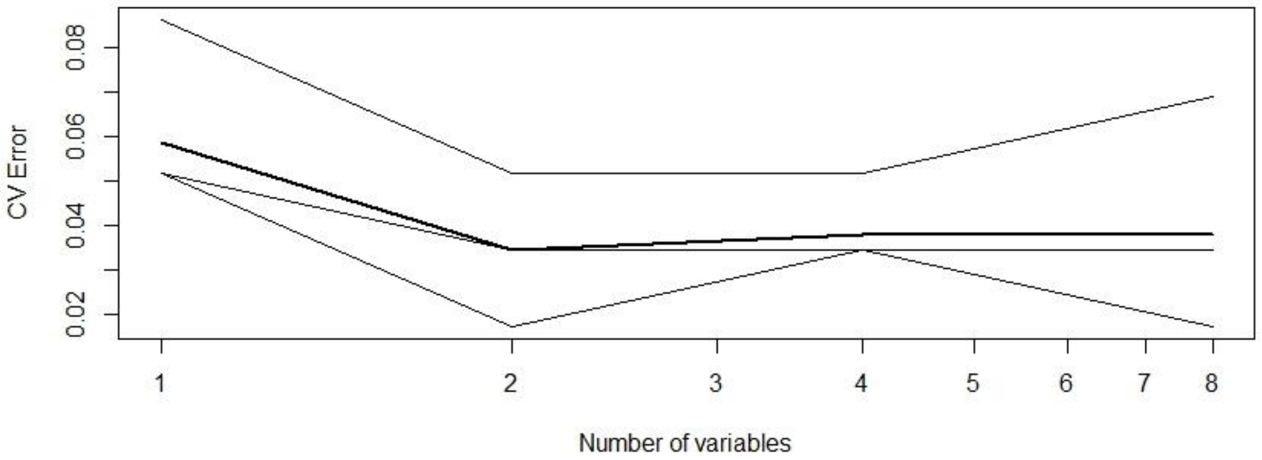

Additional file 03. The best number of variables with the lowest cross validation (CV) error rate is calculated with 5-fold CV in RF.
